# In *vivo* biocompatibility and long-term durability of nanofibrillated cellulose as a urethral bulking agent in rats and Beagle dogs

**DOI:** 10.1371/journal.pone.0317859

**Published:** 2025-02-24

**Authors:** Nina M. M. Peltokallio, Stéphanie Noël, Géraldine Bolen, Satu Kuure, Eija Raussi-Lehto, Guillermo Reyes, Rubina Ajdary, Jani Kuula, Annick Hamaide, Outi M. Laitinen-Vapaavuori

**Affiliations:** 1 Department of Equine and Small Animal Medicine, Faculty of Veterinary Medicine, University of Helsinki, Finland; 2 Teaching and Clinical Department of Companion Animals, Faculty of Veterinary Medicine, University of Liège, Liège, Belgium; 3 GM Unit, Helsinki Institute of Life Science/STEMM, Research Program’s Unit, Faculty of Medicine, University of Helsinki, Helsinki, Finland; 4 Department of Neuroscience and Biomedical Engineering, School of Science, Aalto University, Espoo, Finland; 5 VTT Technical Research Centre of Finland Ltd., Tampere, Finland; 6 Department of Bioproducts and Biosystems, School of Chemical Engineering, Aalto University, Espoo, Finland; Shanghai Jiao Tong University Medical School Affiliated Ruijin Hospital, CHINA

## Abstract

**Background:**

Cystoscopy-assisted submucosal injections of urethral bulking agents offer a safe and efficient alternative to surgery for treating urinary incontinence in both dogs and women. To address the concern of their transient therapeutic effect, a preclinical study evaluating the biocompatibility, safety, and durability of nanofibrillated cellulose as a bulking agent was designed. Plant-based nanofibrillated cellulose is considered renewable, biocompatible, and non-degradable *in vivo*. To the best of our knowledge, no studies of nanofibrillated cellulose injected into the urethral wall of experimental animals have been published to date.

**Methods:**

After assessing the rheological behavior of nanofibrillated cellulose, a biocompatibility study with 50 rats and a durability study with two Beagle dogs were conducted. In anesthesized rats, deposits of either nanofibrillated cellulose or sodium chloride as an inert control were injected into the urethral wall via a caudal laparotomy. The rats were euthanized for histopathological assessment after 7, 30, and 90 days. In dogs, cystoscopy-assisted injections of nanofibrillated cellulose were followed with magnetic resonance imaging at 14 days and at 2, 3, 6, and 12 months.

**Results:**

The rheological studies demonstrated a gel-like behavior under a wide range of shear stress. Nanofibrillated cellulose induced a moderate host tissue response according to the EN ISO 10993-6 standard, consisting primarily of macrophages, foreign body giant cells, lymphocytes, and plasma cells. No significant difference was observed in the tissue response at different time points. In dogs, the bulking agent was visible in 4/5 (80%) injection sites on magnetic resonance imaging at 12 months post-injection. No signs of migration, abscess formation or any major or long-term complications were observed.

**Conclusions:**

Nanofibrillated cellulose maintains a chronic but stable and tolerable inflammatory response for up to 90 days in the urethral wall of rats. Durability in the urethral wall of dogs indicates a potential long-term effect.

## Introduction

Urethral sphincter mechanism incompetence (USMI), a multifactorial condition associated with decreased urethral resistance, is the most common cause of urinary incontinence in neutered female dogs with an incidence of up to 20% [1–3]. In women, the most common cause of urinary incontinence is stress urinary incontinence (SUI). Up to 35% of women experience involuntary leakage of urine during physical activity, sneezing, or coughing caused by a loss of anatomical support, allowing the bladder neck to descend, or weakening of the sphincter muscle, as in USMI [4–7].

Surgical intervention [8–15] or cystoscopy-assisted submucosal injections of urethral bulking agents (UBAs) [16–20] are indicated in dogs refractory to initial medical treatment [21] and in women refractory to pelvic muscle training [22]. UBAs increase urethral resistance by narrowing the urethral lumen, thereby increasing the stretch in sphincter muscle cells and allowing urethral muscles to close more effectively [23]. UBAs have been in clinical use for decades [2,24], more commonly in women as a mini-invasive alternative to surgery with a shorter procedure and recovery time alongside less risks of serious complications [25–28].

Various injectable agents have been used as UBAs [18–20,24,28–33]. Currently, only bovine collagen [18–19] and cross-linked gelatin [20] are on the market for USMI treatment in dogs, and calcium hydroxyl apatite, carbon-coated zirconium, polydimethylsiloxane, and polyacrylamide hydrogel [28,30–33] for SUI treatment in women. A growing interest in UBAs in recent years [25,27] and concerns of their transient therapeutic effect [17–20,33,34] call for more preclinical UBA studies with viable animal models to bridge the gap between *in vitro* studies and clinical use of potential new materials as UBAs in both dogs and women.

In this scenario, cellulose has been demonstrated to be well-suited for biomedical applications due to its renewability, biocompatibility, and cost-effectiveness [35–37]. Furthermore, although cellulose is considered biodegradable in general, it does not degrade *in vivo* due to the lack of cellulase enzymes in animals [38], and as such, has the potential to increase longevity in UBA treatment. Nanofibrillated cellulose (NFC) offers a plant-based alternative [39] to animal-based UBAs, such as bovine collagen and gelatin [18–20], and synthetic materials, such as polyacrylamide and polydimethylsiloxane [28,30–33,40]. NFC is shown to support cell growth [41–43] and promote wound healing [44–46]; however, to the best of our knowledge, no *in vivo* biocompatibility studies of NFC injected to the proximal urethra of experimental animals have been published to date. Hence, a preclinical study with a commercially available NFC (UPM Biomedicals, Helsinki, Finland) was conducted in rats and Beagle dogs.

The aim of this study was to evaluate the biocompatibility, safety, and durability of NFC as a UBA in an experimental animal model. To achieve the objective, the study was divided into two stages: 1) a biocompatibility study in rats and 2) a durability study in Beagle dogs. We hypothesized that NFC would be biocompatible, safe, and durable in the target tissue, and as such, have potential to treat urinary incontinence due to USMI in dogs and SUI in women. Our aim was achieved by demonstrating the host tissue response to NFC in the urethral wall of rats and the durability of NFC in Beagle dogs using repetitive magnetic resonance imaging (MRI) for 12 months.

## Materials and methods

### Materials

In the biocompatibility study, a ready-to-use natural polysaccharide hydrogel-like suspension (UPM Biomedicals, Helsinki, Finland) composed of nanofibrillated cellulose (NFC) gelated in water was used as a urethral bulking agent (UBA) in a solid content of 1.5 wt%. In the durability study, NFC was diluted in MilliQ-water (purified with a Millipore Synergy UV unit, Burlington, MA, USA) down to 1.3 wt% to aid in passing the suspension through a 22G needle. The diluted 1.3% NFC was stored at room temperature and used within 7 days of dilution. Sodium chloride (NaCl, Natriumklorid Braun 9 mg/ml, B. Braun Melsungen AG, Melsungen, Germany) was used as an inert control reagent in the biocompatibility study.

The viscosity of a 1.5% NFC was measured in order to optimize the viscosity needed for a smooth injection procedure and to provide an adequate bulking effect to the urethral wall. Viscosity measurements were conducted using a dynamic rotational rheometer (MCR 203, Anton Paar, Germany), employing parallel plates (PP25) with a 1 mm gap. Viscosity variations were observed in response to increasing shear rates, spanning from 10^−2^ to 10^3^ S^−1^. The linear viscoelastic range was determined through a strain-sweep test, varying from 10^−2^ to 10^2^% at a constant frequency of 10 rad/s, for dynamic viscoelastic assessments. These experiments were all carried out at a controlled temperature of 23°C. Data processing and analysis were executed using the RheoPlus software.

### Animals

#### Ethical approval.

All animal experiments were conducted according to relevant EU and national legislation. Experimental design followed 3R principles [47] and Arrive 2.0 guidelines [48] for reporting the animal experiments. All practical work was carried out by competent persons having the appropriate training and licenses. The study-specific protocol for the biocompatibility study was approved by the Regional State Administrative Agency for Southern Finland (ESAVI/4488/2021), and by the National Animal Ethics Committee of Belgium (ethics agreement no. 2423) for the durability study.

#### Sample size, follow-up, housing of animals.

For the biocompatibility study, a total of 50 healthy 10–12-week-old female Sprague Dawley rats were used, with a follow-up of 7 days (D7 – 20 rats), 30 days (D30 – 15 rats), and 90 days (D90 – 15 rats). Altogether 30 rats were used in the study group (10 rats in each follow-up), and 20 rats in the control group (10 rats at 7 days, 5 rats at 30 days, 5 rats at 90 days). The sample size was calculated based on the 3R principles [47] and a semiquantitative scoring standard for biocompatibility assessment (ISO 10993-6) [49] with a requirement of 10 rats per UBA per time point. The rats were randomly assigned into study or control groups and into different follow-up groups at day 0. They were housed according to the European directive (Directive 2010/63/EU), and Finnish (497/2013) legislation in similar conditions with free access to food, water, and stimuli.

For the durability study, two 7-year-old sexually intact female Beagle dogs, weighing 13.3 and 14.1 kg, were used with a follow-up of 14 days and 2, 3, 6, and 12 months. The sample size was minimized to two dogs based on the 3R principles [47] in order to assess whether the injected NFC is visible in the urethral wall for 12 months. Both dogs were born and housed at the animal facilities of the Teaching and Clinical Department of Companion Animals of the College of Veterinary Medicine, University of Liège, Belgium. The dogs were housed according to the European directive (Directive 2010/63/EU), and Belgian (2010024118) legislation in equal conditions in groups of two in indoor and outdoor runs with exposure to natural light and free access to water. They were fed commercial dry food once a day in amounts sufficient to maintain adequate body weight.

### Biocompatibility study

#### Anesthetic protocol.

**Fig 1:**
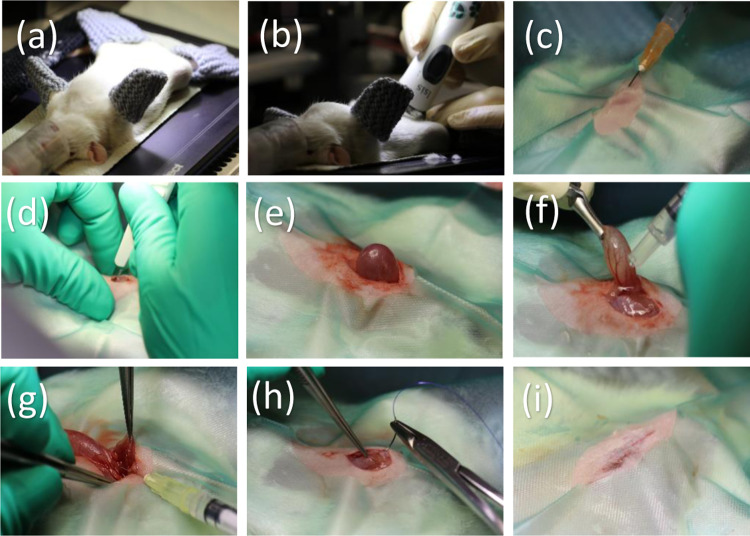
Injecting nanofibrillated cellulose (NFC) into the urethral wall of a rat. (a) An anesthetized rat in dorsal recumbency on a heated plate. (b) The caudal abdomen of the rat is shaved and disinfected. (c) Infiltration of the skin incision with local anesthetic. (d, e) Routine caudal midline abdominal incision to expose the urinary bladder. (f) Local anesthesia to the bladder and proximal urethra. (g) Injection of NFC. (h–i) Routine abdominal closure.

The rats in the D7 follow-up group were premedicated with buprenorphine (0.05 mg/kg subcutaneously) before induction of anesthesia. Each rat was placed in the induction chamber and exposed to 4.5% isoflurane and 600 ml/m of air (Fig 1a). The rats in the D30 and D90 groups were premedicated with a combination of dexmedetomidine (50 µ g/kg), ketamine (5 mg/kg), and buprenorphine (0.015 mg/kg) subcutaneously. Upon complete loss of consciousness, the rats were transferred to a mask with 2.1–2.3% (D7) or 1–1.3% (D30 and D90) isoflurane and 600 ml/m of air (Fig 1a).

#### Surgical procedure.

Rats were placed in dorsal recumbency on a heated plate (+37°C) (Fig 1a). The caudal abdomen was shaved and disinfected with 80% alcohol (Fig 1b). The incision was infiltrated with local anesthetic, 50:50 10% lidocaine, and 5% bupivacaine diluted in sodium chloride (Natriumklorid Braun 9 mg/ml, B. Braun Melsungen AG, Melsungen, Germany) (Fig 1c).

A strict aseptic protocol was followed. A routine caudal midline abdominal incision was made to expose the urinary bladder (Fig 1d and 1e). Local anesthesia with 50:50 10% lidocaine and 5% bupivacaine diluted in sodium chloride (Natriumklorid Braun 9 mg/ml, B. Braun Melsungen AG, Melsungen, Germany) was used as a splash to the bladder and proximal urethra (Fig 1f). The bladder was exteriorized and retracted cranially with an atraumatic, curved DeBakey bulldog clamp to expose the urethra (Fig 1f). Tissues surrounding the proximal urethra were bluntly dissected to expose the injection site as described in Mann-Gow *et al*. (2015) [50]. Two deposits of either 1.5% NFC hydrogel-like suspension (UPM Biomedicals, Finland) or sodium chloride (NaCl, Natriumklorid Braun 9 mg/ml, B. Braun Melsungen AG, Melsungen, Germany) with a volume of 20 µl per deposit were injected into the proximal urethral wall with a 30G needle, in a circumferential arrangement, under direct visualization of an operating microscope (Fig 1g). The injected volume was adjusted to be as small as possible in order not to obstruct the urethral lumen. The abdomen was closed routinely in three layers. Each rat was separately marked for individual identification with a specific combination of earmarks made by an ear punch.

#### Postprocedural monitoring.

After surgery, the rats were monitored and kept on a heated plate (+37°C) until they were fully recovered. For analgesia, the rats received subcutaneously carprofen (5 mg/kg, Rimadyl®, Zoetis Animal Health, Denmark; D7) or meloxicam (1 mg/kg, Metacam®, Labiana Life Sciences S.A., Terrassa, Spain; D30 and D90), a non-steroidal anti-inflammatory drug, before surgery and for two days postoperatively. The rats were checked twice a day for seven days after surgery, and thereafter, once a day until they were euthanized for tissue collection. General behavior and movement were scored on a scale of 0–2 as described in Carstens & Moberg (2000) [51]. Pain was evaluated by the Rat Grimace Scale on a scale of 0–2 [52]. Urination was evaluated by palpation of the urinary bladder twice a day for two days after surgery, and thereafter, by detecting urine in the cage once a day until the rats were euthanized. Wounds were evaluated on a scale of 0–5 daily and photographed when graded abnormal (3–5/5) [53].

#### Macroscopic evaluation and tissue collection.

At the end of each follow-up period, rats were euthanized with CO_2_, followed by decapitation. After euthanasia, the scar and the subcutaneous tissue were evaluated macroscopically for local inflammation, infection, seroma, and/or abscess formation, and the bladder neck and the proximal urethra for possible local inflammation, adhesions, and abscess formation on a scale of 0–1 (0 = absent, 1 = present). The bladder neck, the proximal and mid-urethra, and the bifurcation of the uterus were dissected from the surrounding tissues. Tissue samples were fixed in 4% paraformaldehyde, processed for paraffin with an automated tissue processor (Tissue Tek® VIP, Sakura Finetek, Torrance, CA, USA), and sectioned (Microm cool-cut HM 355S, Thermo Scientific™, USA) longitudinally for histology. A mean of 34 sections per tissue sample were cut to ensure high-quality samples for histopathological analysis. The 5 µm sections were stained with hematoxylin and eosin (H&E) according to standard procedures [54] and assigned a coded identification number.

#### Histopathological scoring according to EN ISO 10993-6 Annex E.

Histopathological assessment was performed by a pathologist (HP) and a veterinary surgeon (NP) in a blinded manner. Possible discrepancies in assessment were resolved by reassessment and discussion. A semiquantitative EN ISO 10993-6 standard [49], designed to test local effects after implantation of biomaterials intended for use in medical devices, was used to analyze the host tissue response and evaluate the biocompatibility of injected UBA. A minimum of two sections per tissue sample were chosen based on the highest quality of the samples, i.e., a maximal amount of injected material visible per high-power (400×) field. The amount and density of polymorphonuclear cells, macrophages, lymphocytes, plasma cells, and foreign body giant cells (FBGCs), and the extent of possible necrosis, neovascularization, fibrosis, and fat reaction were scored on a scale of 0–4 [49]. Due to the greater importance of inflammatory cells and necrosis, possible polymorphonuclear cells, macrophages, lymphocytes, plasma cells, FBGCs, and necrosis present were multiplied by 2 to obtain a point value as compared with neovascularization, fibrosis, and fat reaction [49]. Refer to S1 Table in the Supporting Information for a detailed EN ISO 10993-6 standard.

#### Statistical analysis.

The differences between the three different time points (D7, D30, D90) in the host tissue response were analyzed with a one-way analysis of variance (ANOVA) model, having the day as the sole fixed factor. Least square means were used to estimate the differences between days and within-day averages. The model was fitted for the total score as defined by EN ISO 10993-6 Annex E [49]. The model fit was assessed by evaluating the normality of the studentized model residuals.

### Durability study

#### Procedures.

The dogs underwent a thorough physical examination before inclusion in the study. A vaginal cytologic examination was performed to confirm anoestrus. A blood sample including complete blood count and serum biochemistry was performed, as was a complete urinalysis with bacterial culture obtained via catheterization.

Before the procedures, food was withheld for 12 hours. Dogs were premedicated with an IV bolus of propofol (5–8 mg/kg) and inducted and maintained with a continuous IV infusion of propofol at a dosage sufficient to maintain a stable plane of anesthesia (1–25 mg/kg/h). Both dogs were intubated and monitored with a pulse oximeter and received oxygen supplementation of 2 L/min and continuous IV infusion of Ringer´s lactate (5 ml/kg/h). For analgesia, the dogs received buprenorphine (15 µ g/kg) IV.

The dogs were placed in sternal recumbency, and MRI was conducted under anesthesia before and after contrast medium (gadoteric acid, Clariscan™ 0.5 mmol/ml, GE Healthcare, Chicago, IL, USA, 0.2 ml/kg IV) injection. Dogs were evaluated using a 1.5 Tesla MRI unit (Signa Explorer, GE Medical Systems, Milwaukee, WI, USA) with a 16-channel flex coil. The caudal abdominal protocol comprising the whole urethra and at least the caudal pole of the urinary bladder included transverse (TR: 4057; TE: 101; slice 3 mm), sagittal (TR: 4815; TE: 101; slice 3 mm), dorsal (TR: 5846; TE: 108; slice 3 mm) T2 and 3D sagittal T2 (TR: 2102; TE: 90; slice 1.2 mm), with pre- and post-contrast transverse T1 (TR: 733; TE: 12; slice 3 mm) and transverse LAVA (TR: 10; TE: 3; slice 1 mm) images.

Cystoscopy was subsequently performed in sternal recumbency using a rigid 4 mm cystoscope (Karl Storz, Tuttlingen, Germany). Continuous infusion of sterile 0.9% NaCl maintained bladder and urethral distension. The bladder and the urethra were inspected for any abnormalities. A total of 2–3 deposits (2 to Dog 1, 3 to Dog 2) with a volume of approximately 0.5 ml of 1.3% NFC per deposit were injected in the submucosa of the proximal and mid-urethra with a flexible 22G needle (BoNee®, Coloplast, Minneapolis, MN, USA). The number of deposits and the amount of the injected NFC were adjusted to avoid a significant decrease of the urethral lumen, as would be the case in a clinical setting [16–20]. A postprocedural MRI was conducted as described earlier.

#### Postprocedural monitoring and follow-up evaluation.

Buprenorphine (15 µ g/kg IV) was continued every 12 hours for 2 days and cefazoline (20 mg/kg IV) every 8 hours for 2 days and then every 12 hours for a consecutive 3 days. Dogs were observed until they were completely recovered from anesthesia and monitored for potential complications. They were rechecked for pain and urination every 4 hours on the day of the procedure, thereafter twice daily for 5 days and once daily until the end of the follow-up period. Presence and severity of dysuria, stranguria, hematuria, and length and character of micturition were observed by the examining board-certified veterinary surgeon (SN).

For the follow-up MRIs at 14 days and at 2, 3, 6, and 12 months after the procedure, the dogs were premedicated with methadone (0.1 mg/kg), induced with midazolam (0.2 mg/kg) and propofol (2–3 mg/kg) IV, and maintained with isoflurane (1.5–2%). Both dogs were monitored with a pulse oximeter and received oxygen supplementation of 1 L/min. Measurements of urethral length and diameter, maximal length, height and width (in mm) of the injection sites, and possible signs of substance migration and/or granuloma/abscess formation were assessed from sagittal, dorsal, and transverse MRI images. A board-certified veterinary radiologist (GB) evaluated the images with the Agfa PACS system (Enterprise imaging 8.1.2, AGFA Healthcare).

#### Outcome measures.

The procedure was considered successful if the injected NFC was still visible in MRI images 12 months after the procedure without major complications. Complications were categorized as minor or major using criteria based on Gomes *et al.* (2018) [11]. Minor complications were defined as either self-limiting or those that resolved with conservative therapy. Major complications included those requiring surgery.

## Results

### Rheology

**Fig 2:**
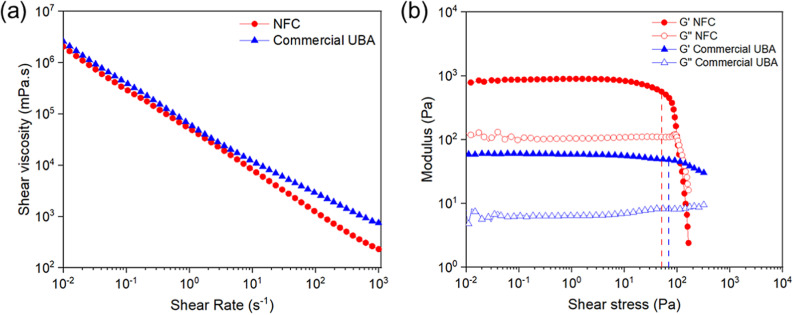
Rheological profile of NFC relative to a commercial UBA, polyacrylamide hydrogel (Bulkamid®, Contura International a/D, Soeborg, Denmark). (a) Flow curves for the apparent viscosity as a function of shear rate and (b) oscillatory rheological behavior of the studied materials. The vertical lines at 50 and 69Pa for NFC and polyacrylamide hydrogel, respectively, represent the critical shear stress values as points where the graph starts to drop due to an increase in the shear stress.

The rheological studies demonstrated similar viscosity profiles of NFC hydrogel-like suspension relative to polyacrylamide hydrogel (Bulkamid®, Contura International A/D, Soeborg, Denmark), a commercially available UBA (Fig 2a). Both NFC and polyacrylamide hydrogel exhibited shear thinning properties in which the viscosity of the hydrogel decreased with the increase of the shear rate. The effect of stress amplitude on the samples is illustrated in Fig 2b. The storage modulus (*G*′)> loss modulus (*G*″) of both samples indicates gel-like behavior and a dominant viscous response under a wide range of shear stresses. The sharp decrease of *G*′ beyond the linear viscoelastic region is due to the structural breakdown of the hydrogel under extensive deformation (Fig 2b) [55,56]. The critical shear stress values at which the ink network displayed a nonlinear viscoelastic behavior corresponded to about 50 Pa and 69 Pa for NFC and polyacrylamide hydrogel, respectively (Fig 2b).

### Biocompatibility study

#### Postprocedural assessment.

All 50 rats survived the study period. Behavior and movement were graded as normal throughout the study. No signs of stranguria or dysuria were observed.

All wounds healed uneventfully either with no interference or with local treatment, with the exception of wound dehiscence due to self-mutilation 1–2 days after surgery in the D7 group (3 rats; 1 NFC, 2 NaCl) and in the D30 group (1 rat; NaCl). After changing the analgesia protocol, only one partial wound dehiscence in a rat injected with NaCl was detected 2 days after surgery.

No signs of abscess or granuloma formation, edema, adhesions, ulceration, or substance spillage or migration were noted at the injection sites at the time of tissue collection. The urethra, urinary bladder, and surrounding tissues were macroscopically normal.

#### Tissue response according to EN ISO 10993-6 Annex E.

**Fig 3:**
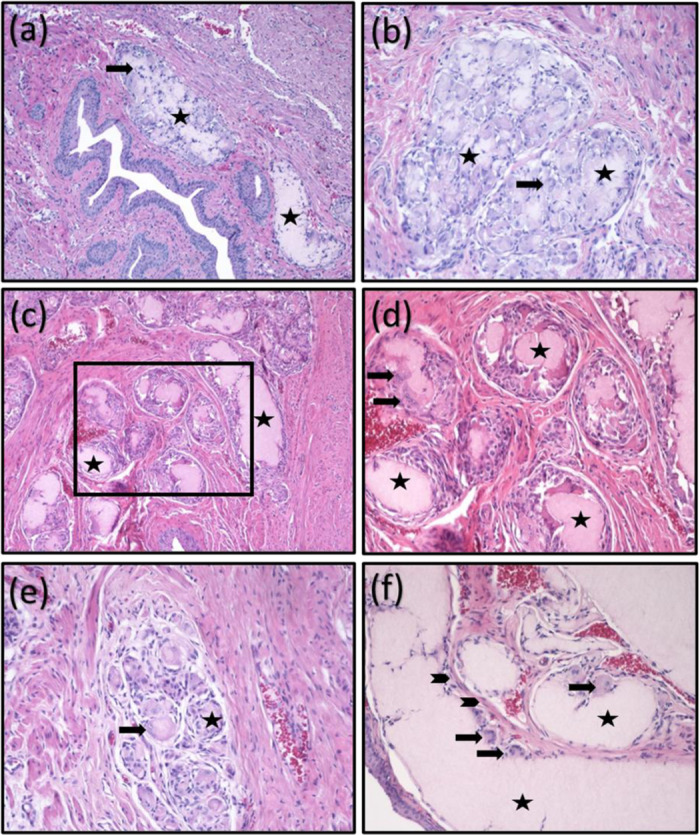
Histopathological analysis of nanofibrillated cellulose (NFC) at D7 (a, b), D30 (c, d), and D90 (e, f). Overview of foreign body reaction surrounding injected NFC. *Black arrows* = foreign body giant cells, *asterisks* = injected NFC, arrowheads = fibroblasts. H&E staining. The boxed area shown in Fig 3c is enlarged in Fig 3d. Bars 400µm (a, c, e) and 200µm (b, d, f).

A total of 50 samples from 50 rats were analyzed. At 7 days, NFC induced a moderate host tissue response (point value 14.7, range 11–19) according to the evaluation criteria of the EN ISO 10993-6 standard used [49] (S2 Table), consisting primarily of abundant macrophages and mild to moderate amounts of FBGCs, lymphocytes, and plasma cells (Table 1). Polymorphonuclear neutrophils were detected in 4/10 study samples (Table 1). The observed host tissue response was localized around the discernible, fragmented NFC deposits with macrophage in-growth (Fig 3). No signs of necrosis, fat reaction, or abscess formation was observed (Table 1).

**Table 1:** Microscopic evaluation of urethral bulking agents (UBAs) composed of nanofibrillated cellulose at D7, D30, and D90 according to EN ISO 10993-6 Annex E [49].

UBA D7 (n = 10)	PNCs^1^	Lymphocytes	Plasma cells	Macrophages	FBGCs^2^	Necrosis	Subtotal (x2)	Neovascularizarion	Fibrosis	Fatty infiltrate	Total	Average (mean ± SD)
1	1	2	1	3	2	0	**18**	1	0	0	**19**	14.7 ± 3.4
2	0	2	2	3	1	0	**16**	1	0	0	**17**	
3	1	1	1	2	1	0	**12**	1	0	0	**13**	
4	1	1	1	3	2	0	**16**	2	0	0	**18**	
5	0	1	1	2	1	0	**10**	1	0	0	**11**	
6	0	1	1	3	0	0	**10**	1	0	0	**11**	
7	1	1	1	3	1	0	**14**	2	0	0	**16**	
8	0	3	2	3	1	0	**18**	1	0	0	**19**	
9	0	1	1	3	1	0	**12**	0	0	0	**12**	
10	0	1	1	2	1	0	**10**	1	0	0	**11**	
**UBA D30 (n = 10)**	**PNCs** ^1^	**Lymphocytes**	**Plasma cells**	**Macrophages**	**FBGCs** ^2^	**Necrosis**	**Subtotal (x2)**	**Neovascularizarion**	**Fibrosis**	**Fatty infiltrate**	**Total**	**Average (mean ± SD)**
11	0	1	0	3	2	0	**12**	2	0	0	**14**	12.4 ± 2.8
12	0	1	1	2	1	0	**10**	1	0	0	**11**	
13	0	1	1	2	1	0	**10**	2	1	0	**13**	
14	0	1	0	3	1	0	**10**	1	0	0	**11**	
15	0	1	1	3	1	0	**12**	1	0	0	**13**	
16	0	2	2	3	1	0	**16**	1	0	0	**17**	
17	1	1	0	2	1	0	**10**	0	0	0	**10**	
18	0	1	0	2	0	0	**6**	1	0	0	**7**	
19	0	1	1	3	2	0	**14**	0	0	0	**14**	
20	0	1	1	3	2	0	**14**	0	0	0	**14**	
**UBA D90 (n = 10)**	**PNCs** ^1^	**Lymphocytes**	**Plasma cells**	**Macrophages**	**FBGCs** ^2^	**Necrosis**	**Subtotal (x2)**	**Neovascularizarion**	**Fibrosis**	**Fatty infiltrate**	**Total**	**Average (mean ± SD)**
21	0	1	1	3	2	0	**14**	1	0	0	**15**	12.1 ± 4.3
22	0	1	1	3	2	0	**14**	1	0	0	**15**	
23	0	1	1	3	1	0	**12**	1	0	0	**13**	
24	0	2	1	3	2	0	**16**	0	0	0	**16**	
25	0	1	0	1	1	0	**6**	2	0	0	**8**	
26	0	1	2	3	2	0	**16**	1	1	0	**18**	
27	0	2	1	2	1	0	**12**	1	0	0	**13**	
28	0	0	0	1	0	0	**2**	2	0	0	**4**	
29	0	1	1	1	0	0	**6**	2	0	0	**8**	
30	0	1	1	2	1	0	**10**	1	0	0	**11**	

Abbreviations:

^1^polymorphonuclear cells,

^2^foreign body giant cells.

The host tissue response assessed by EN ISO 10993-6 Annex E [49] was still moderate 30 days post-injection but had decreased to a point value of 12.4 (range 7–17) (Table 1, S2 Table). The host tissue response observed in the study samples remained consistent with abundant macrophages, mild to moderate cell infiltration of FBGCs, and sporadic lymphocytes and plasma cells, but the initial inflammatory reaction characterized by polymorphonuclear neutrophils had resolved.

At 90 days post-injection, the observed host tissue response remained moderate with a point value of 12.1 (range 4–18) [49] (Table 1, S2 Table), comprising abundant macrophages, mild to moderate cell infiltration of FBGCs, and sporadic lymphocytes and plasma cells similar to 7 and 30 days post-injection. No significant difference was observed in the host tissue response between the different follow-up groups (*p = 0.1608* D7 vs. D30, *p = 0.1147* D1 vs. D90, *p = 0.8522* D30 vs. D90).

The degree of adhesion at different time points was mild with no or a narrow band of single fibroblasts surrounding the NFC deposits (Table 1, Fig 3, S1 Table). Either focal 1–3 buds or groups of 4–7 capillaries with supporting fibroblastic structures were displayed and classified as mild to moderate neovascularization according to EN ISO 10993-6 Annex E [49] at D7, D30, and D90 (Table 1, S1 Table).

Considerable internal variability was noted in the severity of the host tissue response (Table 1, Fig 3) and in the size and shape of the injected NFC deposits between individuals (Fig 3). The host tissue response in the control group was unremarkable. All rats in the control group received 0 points according to EN ISO 10993-6 Annex E [49], indicating no host tissue reaction to sodium chloride at D7, D30, or D90.

A mild to moderate eosinophilic host tissue response was observed in the uterine tissue distant from the injection site at 30 days (8 rats; 4 NFC, 4 NaCl), and 90 days post-injection (9 rats; 5 NFC, 4 NaCl). No such eosinophilic reaction in the uterine tissue was present at 7 days post-injection in either group. The number and severity of eosinophilic reaction were compared between the study and control groups at 30 and 90 days by utilizing a cumulative logit-model, where material and days 30 and 90 were used as fixed factors. The model was constructed to estimate the probability of higher severity of eosinophilic reaction in the uterine tissue. Odds ratios with 95% confidence intervals were used to quantify the differences between injected materials and time points. No significant difference was detected between the study and control groups (*p = 1.1013*, OR 0.291, 0.065–1.297) or between D30 and D90 (*p = 0.8456*, OR 0.873, 0.211–3.607).

### Durability study

#### Procedures.

No abnormalities were found on physical examinations or cystoscopy. Anesthesia was uneventful in both dogs. At the time of injection, blood was detected in the urine sediment of Dog 1, bacterial growth (*Citobacter diversus*) confirmed in bacterial culture and treated according to susceptibility testing. The urinalysis of Dog 2 was unremarkable.

Dilution of NFC to 1.3% resulted in distribution of the suspension along the urethral wall instead of creating submucosal blebs consistent in size and shape. Unintentional trauma to the urethral wall with the cystoscope resulted in superficial mucosal ulcerations in the urethral wall of Dog 1. The procedure with Dog 2 was uneventful.

Dog 1 showed signs of discomfort (dysuria and hematuria) 24 hours after the procedure. Additional pain medication with methadone (0.2 mg/kg IV) and a non-steroidal anti-inflammatory agent (carprofen 2 mg/kg PO) was administered, and the bladder was emptied by manual pressing. Since the symptoms did not resolve within 24 hours, the dog was anesthezised, and a urinary catheter was left in place for 7 days. The dog urinated normally upon removal of the urinary catheter. Dog 2 showed transient hematuria, which resolved without further treatment within 48 hours of the procedure. No long-term complications were observed in either dog during the 12-month follow-up. Both dogs were still asymptomatic at the time of writing the manuscript (30 months post-injection).

#### MRI interpretation.

**Fig 4:**
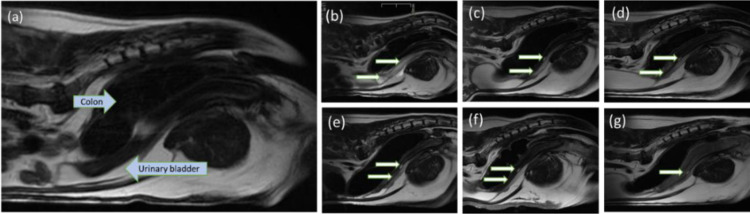
Injected nanofibrillated cellulose (NFC) visible on T2-weighted sagittal MRI images as hyperintense foci in the urethral wall of Dog 2. In the images, cranial is to the left and caudal to the right. *White arrows* = injected NFC. (a) Before injection, (b) immediately after injection, and (c) at 14, (d) at 30, (e) at 90, (f) at 180, and (g) at 365 days post-injection.

**Fig 5:**
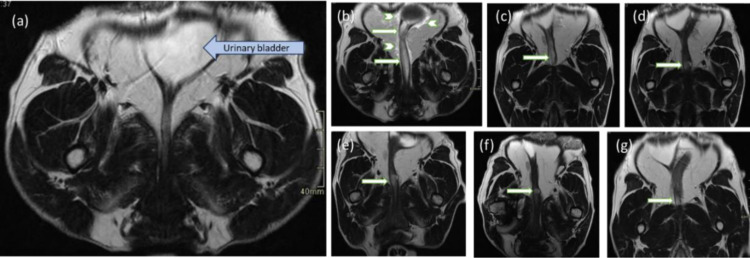
Injected nanofibrillated cellulose (NFC) visible on T2-weighted dorsal MRI images as hyperintense foci in the urethral wall of Dog 2. In the images, the right side of the animal is to the left. *White arrows* = injected NFC. *Arrowhead =* a T2 hyperintense area spreading around the urethra and urinary bladder. (a) Before injection, (b) immediately after injection, and (c) at 14, (d) at 30, (e) at 90, (f) at 180, and (g) at 365 days after injection. The most cranial injection site (b) is poorly visualized on the other acquisition planes (c–g).

The injected NFC was visible on MRI as homogeneous or slightly heterogeneous, hypo- or isointense foci in T1 and hyperintense foci in T2 images compared with the surrounding tissues with no or mild contrast enhancement (Figs 4 and 5). Injection sites ranged from crescent to oval to ill-defined shape. Assessing the three different planes helped delineate the injection sites especially when measuring overlapping foci.

Two different injection sites were visualized in the urethral wall of Dog 1 immediately after UBA injection (Table 2). An oval-shaped, 55 × 5.4 × 6.2 mm T1 hypointense and T2 hyperintense area with no contrast enhancement was detected 27 mm cranial to the urinary meatus. It decreased to a 9 × 1 × 1 mm T1 hypo-/isointense and T2 hyperintense, more ill-shaped area at 12 months. The other, a more crescent-shaped 19 × 6 × 10 mm T1 hypointense and T2 hyperintense area with no contrast enhancement was seen 51 mm cranial to the urinary meatus. It decreased to 13 × 3 × 3 mm at 3 months and was not visible at 6 and 12 months post-injection.

**Table 2:** Width, length, and height measurements (mm) and volume (mm^3^) of the nanofibrillated cellulose (NFC) injection sites on MRI.

	Dog 1 Injection site 1	Injection site 2	Dog 2 Injection site 1	Injection site 2	Injection site 3
**Post-injection**	55 × 5.4 × 6.2 (**1841mm**^**3**^)	19 × 6 × 10 (**1140mm**^**3**^)	14.7 × 7 × 9.8 (**1008mm**^**3**^)	10.7 × 6.2 × 2.4 (**159mm**^**3**^)	48.2 × 5.4 × 6.2 (**1614mm**^**3**^)
**D14**	14 × 2 × 2 (**56mm**^**3**^)	18 × 13 × 20 (**4680mm**^**3**^)	13.8 × 10 × 7 (**966mm**^**3**^)	9 × 2 × 8 (**144mm**^**3**^)	25.4 × 5.4 × 4.7 (**645mm**^**3**^)
**D60**	24 × 2 × 2 (**96mm**^**3**^)	13 × 14 × 10 (**1820mm**^**3**^)	12.7 × 6 × 9 (**686mm**^**3**^)	6 × 6.2 × 2.3 (**86mm**^**3**^)	12 × 3 × 4 (**144mm**^**3**^)
**D90**	19 × 2 × 2 (**76mm**^**3**^)	13 × 3 × 3 (**117mm**^**3**^)	14.7 × 12 × 7 (**1235mm**^**3**^)	6 × 6.6 × 2.3 (**91mm**^**3**^)	26 × 4 × 4 (**416mm**^**3**^)
**D180**	Not visible	Not visible	15.8 × 10.7 × 6.7 (**1133mm**^**3**^)	6 × 7 × 3 (**126mm**^**3**^)	33.4 × 2.7 × 3.3 **(298mm**^**3**^)
**D365**	9 × 1 × 1 (**9mm**^**3**^)	Not visible	17 × 13.4 × 6.7 (**1526mm**^**3**^)	7.6 × 7 × 3 (**160mm**^**3**^)	15 × 1 × 2 (**30mm**^**3**^)

Three different injection sites were visualized in the urethral wall of Dog 2 after UBA injection (Table 2). These were of oval (sites 1 and 3) and crescent shape (site 2). An oval-shaped 14.7 × 7 × 9.8 mm T1 and T2 hyperintense, slightly heterogeneous area with no contrast enhancement on the periphery of the urethra 82 mm to the urinary meatus increased to a more crescent-shaped 17 × 13.4 × 6.7 mm T1 hypointense and T2 iso- to hyperintense, slightly heterogeneous area with slight contrast enhancement and an enhanced thick wall at 12 months. The T1 hyperintensity is expected to originate from mild hemorrhage from the needle tract while injecting. The T2 hyperintense area spreading around the proximal urethra and urinary bladder as shown in Fig 5b was not visible on subsequent imaging from D15. A crescent-shaped 10.7 × 6.2 × 2.4 mm T1 hypointense and T2 hyperintense, homogeneous area with no contrast enhancement 61 mm cranial to the urinary meatus did not show any marked volume reduction at 12 months. On the contrary, an oval-shaped 48.2 × 5.4 × 6.2 mm T1 hypointense and T2 hyperintense, homogeneous area with no contrast enhancement 29 mm cranial to the urinary meatus decreased to a more crescent-shaped 15 × 1 × 2 mm T1 isointense and T2 hyperintense, homogeneous area with mucosal contrast enhancement in 12 months. With the exception of injection sites 1 and 2 in Dog 2, the amount of the injected NFC decreased with time as shown in Table 2. No signs of substance migration or abscess formation were detected.

The urethra of Dog 1 measured 100 mm in length and 5 mm in diameter pre-injection and 102 mm in length and 6.7 mm in diameter 12 months post-injection. At 12 months, a T1 isointense and T2 hypointense periphery of the urethral wall was visualized without contrast enhancement. The urethra of Dog 2 measured 76 mm in length and 6 mm in diameter pre-injection and 120 mm in length and 5.7–9 mm in diameter 12 months post-injection.

## Discussion

The discouraging results with UBAs showing unfavorable short-term effects [17–20,33,34], particle migration [57,58], erosions [59], granuloma [57,60], and pseudoabscess formation [61,62] highlight the need for not only a biocompatible and safe but also a durable UBA to relieve urinary incontinence symptoms. Preclinical studies in viable animal models are a prerequisite before testing potential novel UBAs in clinical trials [63]. *In vitro* assessment of the host immune response to biomaterials cannot replace *in vivo* studies [64]. Only a few *in vivo* UBA biocompatibility studies have been published to date [63,65,66] and none on NFC as a UBA. To the best of our knowledge, this is the first study to demonstrate the biological behavior of NFC in the urethral wall of rats and the durability in the urethral wall of Beagle dogs for 12 months using MRI.

The high water content of hydrogels make them compliant as biomaterials, and as such, impact their ability to provide adequate bulking to the urethral wall to prevent urine leakage [67]. Appropriate viscosity of UBAs is a critical parameter to facilitate a smooth injection process with proper retention [65,68,69], avoiding excessively fluid or thick gels. The NFC hydrogel-like suspension used in this study possesses a non-Newtonian flow with a shear-thinning behavior. Its dynamic viscosity ranges from 10^−1^ Pa⋅s at high shear rates (10^3^ S^−1^) to 10^3^ Pa⋅s at low shear rates (10^−2^ S^−1^) (Fig 2a), allowing an adjustable flow performance by enabling a wide flow operational range and versatility. Importantly, the viscosity and fluid properties of NFC are comparable to commercially available UBAs, such as polyacrylamide hydrogel and calcium hydroxyl apatite [70,71]. In addition to viscosity property *per se*, operation under the linear viscosity region (LVR) is important to maintain the macromolecular structure and stability of the hydrogel, which is critical in maintaining the post-injection solid-like properties and avoiding leakage of the UBA [72]. Higher shear rates or low viscosities ease the injection procedure, as with bovine collagen, but increase the risk of migration or flattening of the injected blebs [73].

The viscosity and flow responses of hydrogels are shear-dependent. In addition, the injection technique, the size and shape of the needle used for injection, and the pressure used while injecting also impact the performance of the UBA chosen [74]. Understanding the typical shear rate is critical since it influences how the hydrogel flows through the needle and how the material behaves upon deposition in the host tissue. However, since the shear rate is determined by the linear syringe speed and cross-section of the needle and since most of the techniques involve manual injection, the surgeon is in control of the linear speed, and consequently, the shear rate [73].

For each new implant, it is essential to understand the host-tissue interaction in response to the different physiochemical properties of the injected material that allow favorable conditions for a safe and durable biomaterial [75,76]. The immune response elicited by the host can be controlled by modulating the surface charge, size, shape, and texture of the implant [64]. A surface charge density of 1.5 mmol/g carboxylate content is typically reached for highly oxidized samples, such as TEMPO-oxidized nanocellulose [77], providing a substantial negative charge density and enhancing dispersibility in water [78]. The morphology of the oxidized nanocellulose fibers ranges from tenths of nanometers in width and several micrometers in length, translating to aspect ratios (L/D) above 100 [78,79]. Thus, combined with the negative fibril charge, the extensive network of hydrogen bonds contributes to flexibility and soft texture, enhancing water dispersibility and promoting gel-like properties. When hydrated, polyacrylamide hydrogel, chemically categorized as a polyolefin, an oil-based polymer, forms soft gels texturally similar to TEMPO-oxidized nanocellulose [80]. Although the polyacrylamide hydrogel itself is considered non-toxic [81], its precursor, acrylamide, is both a neurotoxin and a carcinogen [82]. Consequently, synthetic materials and potential residuals of acrylamide could be prevented with plant-based NFC.

In our study, the semiquantitative ISO 10993-6 assessment of NFC hydrogel-like suspension used as a UBA revealed a consistent, moderate host tissue response characterized mostly by macrophages and mild to moderate amounts of FBGCs. This is an expected and previously reported host tissue reaction to a foreign material [75]. Negligible numbers of lymphocytes and plasma cells were conjointly observed around the deposits. To the best of our knowledge, only one study with a bulking agent injected to the urethral wall of experimental dogs has been published thus far [65]. Sumner *et al.* (2012) [65] investigated the tissue response to polyethylene glycol carboxymethyl cellulose hydrogel (PEG-CMC) and bovine collagen injected to the urethral wall of eight purpose-bred female dogs for 90 days. PEG-CMC incited a granulomatous response comprising a moderately thick layer of macrophages, occasional FBGCs, and rare lymphocytes and plasma cells consistent with the findings in our study. By contrast, collagen incited a lymphoplasmacytic response characterized mostly by lymphocytes and plasma cells with small numbers of histiocytes. The PEG-CMC blebs were macroscopically firm and prominent, whereas the collagen blebs had flattened and elongated. These findings may be of benefit when assessing the longevity of a UBA [65].

In addition, a recent *in vivo* excisional wound healing study with mice investigated the suitability of NFC hydrogel-like suspension for accelerated wound healing over nine days [46]. A mild to moderate inflammatory host tissue reaction with a thin rim of macrophages and single FBGCs was detected in the wounds of 18 mice. Apart from slightly more abundant FBGCs surrounding NFC, the host tissue response detected in our study was similar to that of theirs [46].

Commercial UBAs, such as bovine collagen (ReGain™, Avalon Medical, Stillwater, MN, USA) and gelatin (VetFoam™, BioChange, Yokneam Illit, Israel) used for USMI treatment in dogs, and polydimethylsiloxane (Macroplastique®, Laborie Medical Technologies Corp., Portsmouth, NH, USA) and polyacrylamide hydrogel (Bulkamid®, Contura International A/D, Soeborg, Denmark) used for SUI treatment in women [18–20,28,30–33] induce a milder host tissue reaction than NFC when implanted intraperitoneally, intramuscularly, or subcutaneously to rats and mice [83–86]. This could be of benefit in terms of biocompatibility, but concurrently risk flattening of the blebs [65], and subsequent loss of effect over time. On the contrary, a chronic, granulomatous host tissue reaction could potentially increase longevity, and subsequently, long-term effect for UBA treatment, but also increase risk to negative effects, such as mucosal defects, or abscess formation [59,61,62,65].

Lindner et al. (2022) [86] observed mainly macrophages within the implant beds with low numbers of polymorphonuclear cells, lymphocytes, and fibroblasts after injecting bovine collagen subcutaneously to rats followed for 90 days. FBGCs were observed after material breakdown and cell-ingrowth, as in our study. A similar mild host tissue response was detected within 35 days after injecting gelatin hydrogel subcutaneously to mice [85]. Polydimethylsiloxane induced a mild, but chronic inflammatory reaction with few lymphocytes, macrophages, and fibroblasts when implanted intraperitoneally to rats and intramuscularly to the paravertebral muscles of rabbits [83]. Correspondingly, subcutaneous implantation of polyacrylamide hydrogel led to a minimal inflammatory response, fibrotic encapsulation, and neovascularization in rats [84]. No macrophages, nor cell-infiltration into the hydrogel were detected 8 weeks post-implantation, in contrast to the chronic host tissue response observed with NFC in our study. Similar to our findings, Vasconcelos *et al.* (2015) [87] observed a foreign-body response with FBGCs in the vocal folds of rabbits injected with calcium hydroxyapatite 3 weeks after the procedure. However, since the host tissue reactions were not determined using the same standard as in our study, no direct comparison in relation to the severity of the host tissue response can be made.

With biocompatible materials, acute and chronic inflammatory responses usually resolve within two weeks [75]. NFC seems to maintain a longer chronic inflammatory response for at least up to 90 days post-injection. Macrophages play an essential role not only as inflammatory cells but also in tissue regeneration [75]. Since the initial inflammatory response with polymorphonuclear cells resolved as expected, and no signs of infection, marked fibrosis, or formation of an abscess were detected, we consider the host tissue response in our model tolerable, similar to PEG-CMC findings by Sumner *et al.* (2012) [65]. With the right intensity in the inflammatory response, the foreign body response should outbalance itself over time to reach equilibrium [76]. Since cellulose does not degrade *in vivo* [38], it can be hypothesized that the prolonged host tissue response in our study might lead to increased longevity. However, fragmentation of the NFC deposits, similar to the findings of Koivunotko *et al.* (2024) [46], and the ability of macrophages and FBGCs to degrade the material should be studied further with respect to biodurability. In a drug release study of 20 mice, NFC hydrogel-like suspension remained intact at the injection site for 24 hours [88].

Considerable internal variability in polymorphonuclear cells, lymphocytes, plasma cells, macrophages, and FBGCs was detected between individuals. Such differences in immune reaction can affect the host tissue response [89,90], but it is unlikely to explain the whole underlying pathogenesis behind the milder host tissue response in some individuals even in a homogeneous study group with identical, carefully planned and implemented procedures.

An interesting finding in the rats was a mild to moderate eosinophilic host tissue response in both the study and control samples at 30 and 90 days post-injection. However, the tissue reaction was located distant from the implantation site in the uterine tissue. Eosinophils play a vital role in, e.g., allergic diseases and immune defence against viral, parasitic, and bacterial infections [91,92]. The authors consider the finding irrelevant to the material used since the detected eosinophilic host tissue response was confined to the uterus in both study and control samples and since eosinophils are considered a normal finding in the reproductive tract of cycling rats [93–95]. Our findings underline the importance of a control group in prospective studies.

The injected NFC was visible in all tissue samples after 90 days in rats and in the MRI images of 4/5 (80%) injection sites in Beagle dogs after 12 months. No signs of substance migration were observed. However, 3/5 (60%) injection sites visualized in MRI showed a decrease in the volume of the NFC from post-injection to 12 months. Quantifying the degradation of NFC was challenging due to anatomical differences since the diameter and length of the urethra varied depending on the state of repletion and the position of the bladder in MRI. The dogs were not catheterized for MRI for standard filling of the bladder in order not to cause any damage to the injection sites. The T2 hyperintense area spreading around the proximal urethra and urinary bladder visible in MRI images after injection was expected to be due to overly deep injection of the suspension to the urethral wall. The reaction was not visible from D15, indicating no negative effect. One minor complication (temporary dysuria) in Dog 1 that resolved with urethral catheterization was most likely due to the mucosal ulcerations causing local swelling and pain. No major or long-term complications were observed. Our findings are consistent with the only previously published study with UBAs in continent experimental Beagle dogs over a follow-up of 90 days [65]. The mucosal ulcerations in the urethral wall and the positive bacterial urine culture in Dog 1 highlight the importance of excluding urinary tract infections before the procedure and performing atraumatic procedures.

There were several limitations in our study. In rats, the analgesia protocol was modified between the groups (D7 vs. D30 and D90) due to self-mutilation. Because of the short duration of action of the medications used in this study [96,97], and since no statistical differences were detected between the groups anesthesized with different protocols, those modifications likely did not interfere with the chronic tissue response detected, but instead, emphasize the importance of a multimodal anesthesia [98,99]. The small number of Beagle dogs precludes a true estimation of the long-term behavior of the injected NFC. In clinical patients, distribution of the hydrogel along the urethral wall could lead to flattening of blebs and subsequent loss of effect of the deposits. Additionally, optimizing our MRI technique by placing a urinary catheter might have helped in separating the deposits and delineating the margins better as described in Sumner *et al.* (2012) [65]. Finally, it is important to remember that since the aim of our study was not to significantly decrease the urethral lumen, as it would be in incontinent dogs and women, no conclusions regarding clinical efficacy should be made.

To ease the procedure and to form prominent blebs for a clinical setting, the hydrogel-like suspension used in the current study needs further adjustments. Collecting data over a longer period with dogs affected with urinary incontinence in a clinical setting would provide information on the clinical efficacy and safety of NFC as a UBA. The findings of our study warrant further clinical studies with NFC in incontinent dogs.

## Conclusions

Nanocellulose (NFC) seems to maintain a chronic but stable and tolerable inflammatory response for up to 90 days when injected into the urethral wall of rats. NFC was visible in 4/5 (80%) injection sites on MRI of the two Beagle dogs for 12 months post-injection. No major or long-term complications were observed. Although further clinical studies with longer follow-up periods with dogs affected with USMI are needed, our results show that NFC may potentially provide the desired long-term effect for UBA treatment.

## Supporting information

S1 Table Host tissue response to an implant according to EN ISO 10993-6 Annex E [49]. (DOCX)

S2 Table Severity of host tissue response to an implant according to EN ISO 10993-6 Annex E [49]. (DOCX)
