# Correction: In *vivo* biocompatibility and long-term durability of nanofibrillated cellulose as a urethral bulking agent in rats and Beagle dogs

**DOI:** 10.1371/journal.pone.0327059

**Published:** 2025-06-25

**Authors:** Nina M. M. Peltokallio, Stéphanie Noël, Géraldine Bolen, Satu Kuure, Eija Raussi-Lehto, Guillermo Reyes, Rubina Ajdary, Jani Kuula, Annick Hamaide, Outi M. Laitinen-Vapaavuori

An additional affiliation is missing for the fifth author. Eija Raussi-Lehto is also affiliated with the Future Proof Health and Wellbeing Innovation Hub, Metropolia University of Applied Sciences, Helsinki.
