# Supplementary material for: In vivo biocompatibility and long-term durability of nanofibrillated cellulose as a urethral bulking agent in rats and Beagle dogs
Source: PLoS One. 2025 Feb 24;20(2):e0317859. doi: 10.1371/journal.pone.0317859 (PMC11849868; doi:10.1371/journal.pone.0317859)
Supplement: S2 Table — (DOCX) [file pone.0317859.s002.docx]

***Supporting Information:***

**Table S2: Severity of host tissue response to an implant according to EN ISO 10993-6 Annex E [49].**
